# Income Dynamics and Risk of Colorectal Cancer in Individuals With Type 2 Diabetes: A Nationwide Population-based Cohort Study

**DOI:** 10.2188/jea.JE20230310

**Published:** 2025-01-05

**Authors:** Yong-Moon Mark Park, Benjamin C. Amick, Pearl A. McElfish, Clare C. Brown, Mario Schootman, Marie-Rachelle Narcisse, Seong-Su Lee, Yoon Jin Choi, Kyungdo Han

**Affiliations:** 1 Department of Epidemiology, Fay W. Boozman College of Public Health, University of Arkansas for Medical Sciences, Little Rock, AR, USA; 2 Winthrop P. Rockefeller Cancer Institute, University of Arkansas for Medical Sciences, Little Rock, AR, USA; 3 Department of Internal Medicine, College of Medicine, University of Arkansas for Medical Sciences Northwest, Springdale, AR, USA; 4 Department of Health Policy and Management, Fay W. Boozman College of Public Health, University of Arkansas for Medical Sciences, Little Rock, AR, USA; 5 Department of Psychiatry and Human Behavior, Warren Alpert School of Medicine, Brown University, Providence, RI, USA; 6 Division of Endocrinology and Metabolism, Department of Internal Medicine, College of Medicine, The Catholic University of Korea, Seoul, South Korea; 7 Department of Gastroenterology, National Cancer Center, Goyang, South Korea; 8 Department of Statistics and Actuarial Science, Soongsil University, Seoul, South Korea

**Keywords:** type 2 diabetes, income dynamics, colorectal cancer, risk factor

## Abstract

**Background:**

Individuals with type 2 diabetes mellitus (T2DM) have increased colorectal cancer (CRC) risk, but it is unknown whether income dynamics are associated with CRC risk in these individuals. We examined whether persistent low- or high-income and income changes are associated with CRC risk in non-elderly adults with T2DM.

**Methods:**

Using nationally representative data from the Korean Health Insurance Service database, 1,909,492 adults aged 30 to 64 years with T2DM and no history of cancer were included between 2009 and 2012 (median follow-up of 7.8 years). We determined income levels based on health insurance premiums and assessed annual income quartiles for the baseline year and the four preceding years. Hazard ratios (HRs) and 95% confidence intervals (CIs) were estimated after adjusting for sociodemographic factors, CRC risk factors, and diabetes duration and treatment.

**Results:**

Persistent low income (ie, lowest income quartile) was associated with increased CRC risk (HR_5 years vs 0 years_ 1.11; 95% CI, 1.04–1.18; *P* for trend = 0.004). Income declines (ie, a decrease ≥25% in income quantile) were also associated with increased CRC risk (HR_≥2 vs 0 declines_ 1.10; 95% CI, 1.05–1.16; *P* for trend = 0.001). In contrast, persistent high income (ie, highest income quartile) was associated with decreased CRC risk (HR_5 years vs 0 years_ 0.81; 95% CI, 0.73–0.89; *P* for trend < 0.0001), which was more pronounced for rectal cancer (HR 0.64; 95% CI, 0.53–0.78) and distal colon cancer (HR 0.70; 95% CI, 0.57–0.86).

**Conclusion:**

Our findings underscore the need for increased public policy awareness of the association between income dynamics and CRC risk in adults with T2DM.

## INTRODUCTION

Type 2 diabetes mellitus (T2DM) increases the risk of colorectal cancer (CRC) by about 30%.^1^^,^^2^ Potential mechanisms underlying this increased risk include hyperglycemia, insulin resistance, oxidative stress, and inflammation.^3^ T2DM and CRC also share common lifestyle-related risk factors, such as obesity, physical inactivity, unhealthy diet, and cigarette smoking.^4^

Socioeconomic status (SES), especially income, also contributes to CRC risk. Individuals with low SES are less likely to engage in healthy behaviors and participate in a CRC screening.^5^^–^^7^ While low SES is associated with an increased CRC risk in the United States and Canada,^8^^,^^9^ studies in European and Asian countries often find no association or even a decreased CRC risk associated with low SES.^8^^–^^10^ Because most studies have only assessed income at one point in time and ignored the possible effects of income changes,^8^^,^^9^^,^^11^^–^^16^ strong conclusions on the association between individual income status and CRC risk remain inconclusive.^11^^–^^16^ Some recent studies have investigated CRC risk in relation to the group- or area-level income status over time, but this approach may not accurately reflect individual income change.^15^^,^^17^^–^^21^ This method may underestimate the importance of individual income dynamics on CRC incidence by ignoring the cumulative effects of individual income changes over time. Changes in income, especially declines, may increase CRC risk in individuals with T2DM through changes in lifestyle risk factors and financial barriers that limit access to routine CRC screening and management of T2DM and its complications.^22^^,^^23^

In this study, we examined whether persistent low or high income and changes in income are associated with the risk of CRC development in individuals with T2DM using data from South Korea’s National Health Insurance System (NHIS), which covers 97% of the country’s population.

## METHODS

### Data source

The NHIS captures medical claims for the entire South Korean population. This includes the 3% of the population covered by the Medical Aid Program, which provides medical benefits to those with the lowest income level. Details of the NHIS database are described elsewhere.^24^^,^^25^ Briefly, the NHIS database contains sociodemographic and claims data, such as diagnosis, medical procedures, prescriptions, and hospital admission. Furthermore, the NHIS provides standardized national health screening examinations and collects data on health behaviors, anthropometric measurements, and laboratory tests. The NHIS database has been validated for research purposes.^26^^,^^27^ This study was approved by the Institutional Review Board of Soongsil University (SSU-202003-HR-201-01) and complied with the ethical guidelines of the World Medical Association Declaration of Helsinki.

### Study population

**Figure 1.**
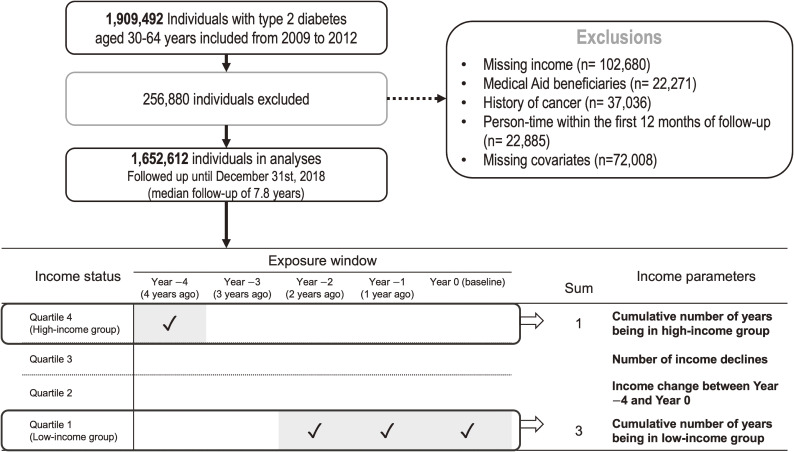
Study population flow diagram and income parameters. After applying exclusion criteria, 1,652,612 subjects were included in the final analysis. The cumulative number of years in the low- or high-income group was counted for the baseline year and the preceding 4 years. Income decline was defined as a decrease of ≥25% in income compared with the previous year’s income, with the 20 quantile-level health insurance premiums analyzed as a continuous variable. Changes in income status (ie, rise and decline) were compared between the first (4 years before baseline) and the last (baseline) time points.

We included 1,909,492 individuals with T2DM aged 30–64 years who underwent national health screening examinations between 2009 and 2012 (index years). For each study subject, the index year (or baseline year) was determined as the year they received their initial health screening examination within this timeframe. The study was limited to this age group because they were considered economically active. Individuals with T2DM were defined as having at least one claim per year for a history of prescribed oral antidiabetic medication or insulin (International Classification of Diseases, Tenth Revision [ICD-10] codes E11–E14^24^) or a fasting blood glucose level ≥126 mg/dL in the national health screening examinations. Individuals with type 1 diabetes (E10) or gestational diabetes (O24) were not included. We excluded those with missing income data (*n* = 102,680), Medical Aid beneficiary status (*n* = 22,271), a previous history of cancer by identifying claims related to cancer diagnoses since 2005 (*n* = 37,036), or with at least one missing covariate (*n* = 72,008) at baseline. We did not include Medical Aid beneficiaries due to their heterogeneous nature compared to other groups enrolled in NHIS.^28^^,^^29^ After further excluding person-times within the first year of follow-up to reduce the bias related to undetected CRC present at baseline (*n* = 22,885), 1,652,612 subjects were included in the analysis (Figure 1). The study population was followed until December 31st, 2018. Individuals were censored at the development of colorectal cancer, last follow-up, or death, whichever occurred first. Deaths from other causes were treated as censoring events.

### Ascertainment of income exposure

Income is the primary factor in calculating NHIS premiums.^30^ While actual household income data are not available from the NHIS database, health insurance premiums are determined as 20 quantile levels by wage income among government and school employees and industrial workers (employee insured), and income and property among the self-employed (self-employed insured).^30^ Thus, we used monthly health insurance premiums as a surrogate for household incomes. The monthly insurance premium remains fixed across a 12-month period unless there is a sudden, drastic income change.^31^ We defined income for each individual by collecting their health insurance premium data which were presented in 20 quantiles annually during a retrospective, 5-year span that included their baseline year (eg, we collected insurance premium data from 2005 through 2009 for those who completed the national health screening in 2009). We used the following approaches to define income exposure:

*Cumulative number of years being in the low- or high-income groups:* We calculated two income mobility metrics: the cumulative number of years in the lowest income quartile and the cumulative number of years in the highest income quartile. Specifically, we determined income quartiles for each year during the 5-year period (consisting of 4 years pre-baseline and the baseline year), and then computed the total number of years an individual belonged to the highest and lowest income quartiles to assess each individual’s income pattern.^32^^,^^33^ For example, if an individual was in the lowest quartile of income for 3 of 5 years, their ‘cumulative years of being in the low-income group’ value was marked as 3. We followed the same process for the ‘cumulative years of years being in the high-income group’ (Figure 1).

*Number of income declines:* Income decline was defined as a reduction of ≥25% in income level^34^^,^^35^ from one year to the next. To calculate the degree of reduction, we computed the difference between income levels in adjacent years and divided it by the first of the 2 years. For instance, if an individual was in the 20th quantile of health insurance premiums in 2008 and moved to the 15th quantile in 2009, that individual was noted as experiencing a 25% reduction in income level. The categories ‘0,’ ‘1,’ and ‘≥2’ indicate the number of qualifying (ie, ≥25%) income declines experienced by individuals during the 5-year period. Individuals in category “0” experienced no such declines. Those in category “1” experienced a single income decline of ≥25%, while those in “≥2” experienced two or more declines.

*Other measures of income status:* Individuals’ baseline income status was categorized into quartiles. In addition, changes in income status (ie, rise and decline) were compared between the first time point (4 years before baseline) and the last (baseline).

### Ascertainment of CRC

The primary outcome was incident CRC, based on ICD-10 codes (C18.0–18.4 for proximal colon cancer; C18.5–18.7, C19.0 for distal colon cancer; C20.0 for rectal cancer, and V193, the registration code for cancer). The NHIS has applied special reimbursement codes (V codes) for intractable diseases such as cancers since 2006. Reimbursement of cancer-related management requires a certified diagnosis by a physician and the medical institution, ensuring that CRC diagnoses based on both C and V codes are highly accurate. In a subgroup analysis by tumor subsite, we excluded malignant neoplasms in overlapping sites in the colon (C18.8) and unspecified colon cancer (C18.9).

### Measurement of covariates

Information in the NHIS database on lifestyle-related factors was obtained using self-administered questionnaires. The cumulative lifetime smoking exposure was calculated as the pack years by multiplying the average daily number of cigarettes smoked (pack) by the total duration of smoking (years). Alcohol consumption was also categorized into non-drinking, mild-to-moderate drinking (<30 g/day), and heavy drinking (≥30 g/day). Regular exercise was defined as at least 30 minutes of moderate-intensity physical activity for ≥5 days weekly or at least 20 minutes of strenuous physical activity ≥3 days weekly.

The anthropometric and clinical characteristics were assessed by trained personnel during the health screening examination. Body mass index (BMI) was calculated as body weight divided by the square of height (kg/m^2^). Abdominal obesity was defined as a waist circumference ≥90 cm for men and ≥85 cm for women, according to South Korean population standards and guidelines.^36^ Systolic and diastolic blood pressures were measured after at least 5 minutes of rest with the patient in a sitting position. Blood samples were collected after overnight fasting, and serum levels of glucose and creatinine were measured. Quality control procedures for laboratory tests were performed based on the Korean Association of Laboratory Quality Control guidelines.

The presence of hypertension was defined as having at least one prescription of anti-hypertensive medications under ICD-10 codes I10–I15 per year or systolic/diastolic blood pressure ≥140/90 mm Hg. Chronic kidney disease was defined as an estimated glomerular filtration rate <60 mL/min. Other clinical comorbidities were defined using the ICD-10 codes and the prescription lists in the NHIS database. Newly diagnosed diabetes was defined as having no prior history of claims for ICD-10 codes for diabetes (E11–E14) or antidiabetic medication claims in the NHIS database and a fasting glucose level ≥126 mg/dL at baseline.

### Statistical analysis

Baseline characteristics were presented as counts and percentages or as means with standard deviations. The CRC incidence rate was calculated by dividing the number of events by the total number of person-years of follow-up presented per 1,000 person-years.

Cox proportional hazard models were used to estimate hazard ratios (HRs) and 95% confidence intervals (CIs) for the association between different income status indices and CRC risk. The proportional-hazards assumption was evaluated using the Schoenfeld residuals test, with the logarithm of the cumulative hazards function based on Kaplan–Meier estimates for various income parameters of interest. There was no significant departure from proportionality in hazards over time. Tests for linear trends were analyzed with linear regression, with the ordinal number assigned to each income category as the predictor variable. We adjusted for potential confounders at baseline using four different models. In addition to an unadjusted model (model 1), model 2 adjusted for age and sex. Model 3 further adjusted for pack-years of smoking, alcohol consumption, physical activity, income quantile at 4 years pre-baseline, residential location, and health insurance type to account for lifestyle and sociodemographic characteristics. In model 4, we additionally adjusted for metabolic CRC risk factors and diabetes status and management, including BMI, abdominal obesity, hypertension, chronic kidney disease, use of statin and aspirin, as well as the blood glucose concentrations, T2DM duration, oral anti-diabetic medications prescribed per year, and history of insulin prescription.

We also calculated fully adjusted HR separately by anatomic sites of CRC (ie, proximal colon, distal colon, and rectum), as the association between SES and CRC risk may differ by anatomic location.^37^^,^^38^ In supplemental analyses, we assessed the potential effect modification by age group, sex, obesity, smoking, alcohol consumption, regular exercise, residential area, insurance type, BMI, and T2DM duration using stratified analyses and tests for interaction based on likelihood ratio tests. We also conducted a sensitivity analysis with a 5-year landmark point to estimate the unbiased CRC risk in those who were event-free until 5 years of follow-up, examining the long-term association between income exposures and CRC risk.^39^

Statistical analyses were performed using SAS version 9.4 (SAS Institute Inc., Cary, NC, USA). The *P* values provided are two-sided, with the level of significance at 0.05.

## RESULTS

Study population baseline characteristics are shown in Table 1, stratified by the cumulative years of being in low- or high-income groups in individuals with T2DM. Individuals who were continuously in the low-income group (*n* = 5 years) were more likely to be older, female, nonsmokers, nondrinkers, physically inactive, not overweight (BMI <23 kg/m^2^), and were less likely to be self-employed insured or live in a rural area than those who never experienced a low-income status (*n* = 0 years). Individuals in this low-income group were also more likely to have abdominal obesity, hypertension, chronic kidney disease, and a longer duration of T2DM (≥5 years), more likely to use statins, aspirin, oral anti-diabetic medications prescribed per year (at least three), and insulin treatment. In contrast, those who were continuously high-income, compared to those who were never high-income, showed the opposite trend in health insurance type and positive trends in smoking, drinking, and physical activity. Characteristics by income quartiles at baseline years ranging from 2009 to 2012 are shown in eTable 1.

**Table 1.** Baseline characteristics by cumulative years being in low- or high-income group in adults with type 2 diabetes

Baseline characteristics	Total population	Cumulative number of years being in low-income group^a^			Cumulative number of years being in high-income group^a^		
	
		0	1–4	5	0	1–4	5
*N*	1,652,612	1,025,648	514,681	112,283	1,481,509	132,369	38,734
**Percent (%)**							
Sex							
Male	65.8	69.7	59.7	58.2	65.2	69.1	74.9
Female	34.2	30.3	40.4	41.9	34.8	30.9	25.1
Age group, years							
<45	9.6	9.5	10.6	5.4	10.3	4.4	0.9
45–<55	49.7	51.6	46.8	46.1	49.7	49.9	52.0
≥55	40.7	38.9	42.7	48.5	40.1	45.7	47.2
Health insurance type							
Self-employed insured	32.3	36.4	25.5	26.5	31.4	38.5	44.3
Employee insured	67.7	63.6	74.5	73.5	68.6	61.5	55.7
Residential place							
Metropolitan	59.5	59.8	58.7	60.3	58.5	66.4	71.1
Urban	29.5	29.3	30.0	28.6	30.0	26.2	22.9
Rural	11.1	10.9	11.4	11.2	11.5	7.4	6.0
Smoking, pack-years							
Never	49.3	47.2	52.7	53.1	49.3	49.3	48.3
<10	11.2	11.7	10.5	8.9	11.2	10.8	10.7
10–<20	14.0	15.0	12.5	11.2	14.0	13.5	13.6
≥20	25.6	26.1	24.3	26.8	25.5	26.5	27.4
Alcohol consumption							
Non	49.0	46.7	52.3	54.6	49.2	47.7	45.1
Mild to moderate	38.6	40.3	36.1	34.4	38.3	39.9	42.5
Heavy	12.5	13.0	11.6	11.0	12.5	12.4	12.4
Regular exercise, yes^b^	20.8	21.9	19.1	19.5	20.3	25.2	27.1
Body mass index, kg/m^2^							
<18.5	1.2	1.0	1.4	1.5	1.2	0.7	0.6
18.5–<23	23.2	22.4	24.5	25.3	23.4	21.4	21.2
23–<25	24.7	25.0	24.2	24.1	24.5	26.0	27.5
25–<30	42.6	43.6	41.2	40.9	42.4	44.6	44.7
≥30	8.3	8.1	8.8	8.2	8.5	7.4	6.1
Abdominal obesity, yes	36.0	36.0	35.8	36.8	35.9	36.8	35.7
Hypertension, yes	48.9	48.3	49.2	52.3	48.8	49.3	50.1
Hypercholesterolemia, yes	39.1	39.3	38.6	39.7	38.5	43.5	45.3
Chronic kidney disease, yes	7.0	7.1	6.6	7.5	6.8	8.3	7.7
Statin use, yes	26.6	26.8	26.1	26.8	25.9	32.0	34.3
Aspirin use, yes	22.4	22.3	22.3	23.7	22.0	25.8	27.7
Oral anti-diabetic medications prescribed per year (at least 3)	12.5	12.3	12.8	12.9	12.4	13.1	13.1
Insulin treatment, yes	6.3	6.1	6.6	6.5	6.2	6.6	6.4
Type 2 diabetes duration							
Newly diagnosed	49.6	49.6	49.5	49.7	50.4	43.4	41.1
<5 years	25.6	25.6	25.7	26.1	25.4	27.7	27.9
≥5 years	24.8	24.8	24.8	24.3	24.2	29.0	31.0
**Mean (standard deviation)**							
Age, years	51.6 (8.5)	51.4 (8.4)	51.8 (8.8)	53.4 (7.8)	51.4 (8.6)	53.1 (7.6)	54.0 (6.4)
Body mass index, kg/m^2^	25.3 (3.4)	25.3 (3.3)	25.2 (3.5)	25.1 (3.5)	25.3 (3.4)	25.3 (3.1)	25.2 (3.0)
Waist circumference, cm	85.3 (8.6)	85.6 (8.4)	84.9 (8.8)	85.0 (8.8)	85.3 (8.6)	85.8 (8.3)	85.9 (8.1)
Fasting glucose, mg/dL	140.7 (49.6)	140.1 (48.1)	142.0 (51.6)	141.3 (53.2)	140.9 (50.2)	139.5 (44.4)	137.9 (41.6)
Systolic blood pressure, mm Hg	128.0 (15.5)	127.9 (15.3)	128.2 (15.9)	128.7 (16.2)	128.2 (15.6)	126.8 (15.0)	125.9 (14.5)
Diastolic blood pressure, mm Hg	79.6 (10.3)	79.6 (10.2)	79.5 (10.4)	79.8 (10.5)	79.7 (10.3)	78.8 (10.0)	78.5 (9.8)
Total cholesterol, mg/dL	199.4 (42.1)	199.2 (41.8)	199.8 (42.4)	200.5 (43.1)	199.8 (42.1)	196.9 (41.6)	195.0 (40.7)

Data are presented as percentages for categorical variables and means (standard deviation) for continuous variables.

^a^The number of times an individual was categorized in the low- or high-income group was counted annually for the baseline year (2009–2012) and the preceding four years.

^b^Regular exercise was defined as at least 30 minutes of moderate-intensity physical activity for 5 or more days per week, or at least 20 minutes of strenuous physical activity for 3 or more days per week.

Associations between various income dynamics indicators and CRC risk are shown in Table 2. A total of 21,978 incident CRC cases were identified at least 1 year after baseline during a median of 7.8 (interquartile range, 7.0–8.3) years of follow-up. Those who were consistently in low-income status had the highest increased risk of CRC (model 4: HR 1.11; 95% CI, 1.04–1.18; *P* for trend = 0.004), compared with those who had never been in the low-income group. Compared with individuals with no income declines, those with ≥2 declines had a greater risk of CRC (model 4: HR 1.10; 95% CI, 1.05–1.16; *P* for trend = 0.001). Those who were consistently in high-income status had a decreased risk with the largest magnitude (model 4: HR 0.81; 95% CI, 0.73–0.89; *P* for trend < 0.0001), compared with those who had never been in the high-income group. Higher income at baseline was associated with decreased risk of CRC, although the strength of association was not considerable (model 4: HR_highest vs lowest quartile_ 0.90; 95% CI, 0.86–0.94; *P* for trend < 0.0001) compared to that of the cumulative measure of high-income status (Table 2 and eTable 2).

**Table 2.** Association between income dynamics indicators and colorectal cancer risk in adults with type 2 diabetes

	Number of participants	Number of events	Total number of person-years of follow-up	Incidence rate (per 1,000 person-years)	Risk difference (95% CI)	Model 1	Model 2	Model 3	Model 4
						HR (95% CI)	HR (95% CI)	HR (95% CI)	HR (95% CI)
Cumulative number of years being in low-income group									
0	1,025,648	13,153	7,646,477	1.72	1 (Ref.)	1 (Ref.)	1 (Ref.)	1 (Ref.)	1 (Ref.)
1–4	514,681	6,973	3,787,012	1.84	0.03 (0.00–0.06)	1.07 (1.04–1.10)	1.06 (1.03–1.09)	1.03 (1.00–1.07)	1.03 (1.00–1.07)
5	112,283	1,852	832,663	2.22	0.08 (0.04–0.15)	1.29 (1.23–1.36)	1.17 (1.11–1.23)	1.11 (1.05–1.18)	1.11 (1.04–1.18)
*P* for trend						<0.0001	<0.0001	0.003	0.004
Number of income declines									
0	1,044,797	13,621	7,800,539	1.75	1 (Ref.)	1 (Ref.)	1 (Ref.)	1 (Ref.)	1 (Ref.)
1	485,639	6,574	3,570,677	1.84	0.01 (−0.02 to 0.03)	1.06 (1.03–1.09)	1.02 (0.99–1.05)	1.01 (0.98–1.04)	1.01 (0.98–1.04)
≥2	122,176	1,783	894,936	1.99	0.07 (0.04–0.13)	1.14 (1.09–1.20)	1.10 (1.05–1.16)	1.11 (1.05–1.16)	1.10 (1.05–1.16)
*P* for trend						<0.0001	0.0004	0.0004	0.001
Cumulative number of years being in high-income group									
0	1,481,509	19,793	11,003,701	1.80	1 (Ref.)	1 (Ref.)	1 (Ref.)	1 (Ref.)	1 (Ref.)
1–4	132,369	1,720	977,148	1.76	−0.08 (−0.12 to −0.04)	0.98 (0.93–1.03)	0.87 (0.83–0.92)	0.90 (0.86–0.95)	0.91 (0.86–0.96)
5	38,734	465	285,302	1.63	−0.17 (−0.25 to −0.10)	0.91 (0.83–0.99)	0.76 (0.70–0.84)	0.80 (0.73–0.88)	0.81 (0.73–0.89)
*P* for trend						<0.0001	<0.0001	<0.0001	<0.0001
Baseline income									
Quartile 1	339,053	5,088	2,496,142	2.04	1 (Ref.)	1 (Ref.)	1 (Ref.)	1 (Ref.)	1 (Ref.)
Quartile 2	347,270	4,590	2,558,400	1.79	−0.04 (−0.08 to −0.01)	0.88 (0.85–0.92)	0.95 (0.92–0.99)	0.95 (0.91–0.99)	0.95 (0.92–0.99)
Quartile 3	452,585	5,750	3,376,521	1.70	−0.07 (−0.11 to −0.04)	0.84 (0.80–0.87)	0.91 (0.88–0.95)	0.92 (0.88–0.95)	0.92 (0.88–0.96)
Quartile 4	513,704	6,550	3,835,089	1.71	−0.09 (−0.13 to −0.05)	0.84 (0.81–0.87)	0.87 (0.84–0.90)	0.89 (0.85–0.93)	0.90 (0.86–0.94)
*P* for trend						<0.0001	<0.0001	<0.0001	<0.0001

CI, confidence interval; HR, hazard ratio.

Quartile 1 represents the lowest income category, while quartile 4 represents the highest income category.

Model 1: unadjusted. Model 2: adjusted for age and sex. Model 3: adjusted for model 2 covariates, plus pack-years of smoking (never, <10, 10 to 19, or ≥20 years), alcohol consumption (never, mild to moderate, or heavy), physical activity (regular exercise or not), income 20-quantile (based on income level 4 years before baseline), residential location (metropolitan, urban, or rural), and health insurance type (employee insured, self-employed insured, or Medical Aid). Model 4: adjusted for model 3 covariates, plus body mass index (continuous), presence of abdominal obesity, hypertension, chronic kidney disease, use of statin and aspirin, blood glucose concentrations, diabetes duration (newly diagnosed type 2 diabetes, <5 years, or ≥5 years), number of oral anti-diabetic medication prescriptions per year (<3, or ≥3), and history of insulin prescription.

Associations between income dynamics indicators and CRC risk by anatomical tumor location are shown in Table 3. The association between cumulative years in low-income status and CRC risk was significant only for distal colon cancer (HR_5 years vs 0 years_ 1.15; 95% CI, 1.02–1.29). Individuals with ≥2 declines had a significantly increased risk of distal colon and rectal cancers (HR 1.15; 95% CI, 1.05–1.27; *P* for trend = 0.01 and HR 1.16; 95% CI, 1.06–1.27; *P* for trend = 0.004, respectively). In contrast, the associations of cumulative number of years in high-income status and baseline income with CRC risk were statistically significant and more pronounced for distal colon and rectal cancers (HR_5 years vs 0 years_ 0.70 and 0.64, respectively; HR_highest vs lowest quartile_ 0.81 and 0.85, respectively) than for proximal colon cancer (HR_5 years vs 0 years_ 0.79; HR_highest vs lowest quartile_ 0.94).

**Table 3.** Association between income dynamics indicators and colorectal cancer risk in adults with type 2 diabetes by tumor location

	Proximal colon cancer				Distal colon cancer			
	
	Number of events	Incidence rate (per 1,000 person- years)	Risk difference (95% CI)	Adjusted HR (95% CI)	Number of events	Incidence rate (per 1,000 person- years)	Risk difference (95% CI)	Adjusted HR (95% CI)
Cumulative number of years being in low-income group								
0	1,467	0.19	1 (Ref.)	1 (Ref.)	3,317	0.43	1 (Ref.)	1 (Ref.)
1–4	760	0.20	0.00 (−0.02 to 0.01)	0.99 (0.89–1.10)	1,853	0.49	0.01 (−0.02 to 0.03)	1.04 (0.98–1.12)
5	212	0.25	0.01 (−0.01 to 0.03)	1.11 (0.93–1.33)	533	0.64	0.03 (0.00–0.06)	1.15 (1.02–1.29)
*P* for trend				0.37				0.07
No. of income declines								
0	1,506	0.19	1 (Ref.)	1 (Ref.)	3,480	0.45	1 (Ref.)	1 (Ref.)
1	723	0.20	0.00 (−0.01 to 0.01)	0.99 (0.91–1.08)	1,744	0.49	0.01 (−0.01 to 0.02)	1.04 (0.98–1.10)
≥2	210	0.23	0.01 (0.00–0.03)	1.15 (0.99–1.33)	479	0.53	0.03 (0.01–0.06)	1.15 (1.05–1.27)
*P* for trend				0.14				0.01
Cumulative number of years being in high-income group								
0	2,205	0.20	1 (Ref.)	1 (Ref.)	5,218	0.47	1 (Ref.)	1 (Ref.)
1–4	181	0.19	−0.02 (−0.03 to 0.00)	0.83 (0.71–0.97)	381	0.39	−0.06 (−0.07 to −0.03)	0.79 (0.70–0.88)
5	53	0.19	−0.02 (−0.04 to 0.00)	0.79 (0.60–1.05)	104	0.36	−0.07 (−0.10 to −0.04)	0.70 (0.57–0.86)
*P* for trend				0.03				<0.0001
Baseline income								
Quartile 1	563	0.23	1 (Ref.)	1 (Ref.)	1,429	0.57	1 (Ref.)	1 (Ref.)
Quartile 2	498	0.19	0.00 (−0.03 to 0.01)	0.96 (0.85–1.08)	1,215	0.47	−0.02 (−0.04 to 0.00)	0.91 (0.84–0.98)
Quartile 3	640	0.19	−0.04 (−0.05 to −0.02)	0.95 (0.84–1.06)	1,475	0.44	−0.04 (−0.05 to −0.02)	0.86 (0.80–0.93)
Quartile 4	738	0.19	−0.05 (−0.07 to −0.03)	0.94 (0.82–1.07)	1,584	0.41	−0.05 (−0.07 to −0.03)	0.81 (0.74–0.88)
*P* for trend				0.76				<0.0001

	Rectal cancer							

	Number of events	Incidence rate (per 1,000 person- years)	Risk difference (95% CI)	Adjusted HR (95% CI)				
Cumulative number of years being in low-income group								
0	3,866	0.50	1 (Ref.)	1 (Ref.)				
1–4	2,146	0.56	0.01 (−0.01 to 0.03)	1.04 (0.98–1.11)				
5	559	0.67	0.01 (−0.02 to 0.04)	1.05 (0.94–1.17)				
*P* for trend				0.42				
Number of income declines								
0	4,034	0.52	1 (Ref.)	1 (Ref.)				
1	1,984	0.55	0.01 (−0.01 to 0.02)	1.03 (0.98–1.09)				
≥2	553	0.62	0.04 (0.02–0.07)	1.16 (1.06–1.27)				
*P* for trend				0.004				
Cumulative number of years being in high-income group								
0	6,002	0.54	1 (Ref.)	1 (Ref.)				
1–4	464	0.47	−0.04 (−0.06 to −0.02)	0.86 (0.78–0.95)				
5	105	0.37	−0.10 (−0.14 to −0.06)	0.64 (0.53–0.78)				
*P* for trend				<0.0001				
Baseline income								
Quartile 1	1,545	0.62	1 (Ref.)	1 (Ref.)				
Quartile 2	1,464	0.57	0.00 (−0.02 to 0.02)	0.99 (0.92–1.06)				
Quartile 3	1,742	0.51	−0.02 (−0.04 to 0.00)	0.92 (0.86–0.99)				
Quartile 4	1,820	0.47	−0.04 (−0.07 to −0.02)	0.85 (0.78–0.92)				
*P* for trend				0.0002				

CI, confidence interval; HR, hazard ratio.

Quartile 1 represents the lowest income category, while quartile 4 represents the highest income category.

Model 1: unadjusted. Model 2: adjusted for age and sex. Model 3: adjusted for model 2 covariates, plus pack-years of smoking (never, <10, 10 to 19, or ≥20 years), alcohol consumption (never, mild to moderate, or heavy), physical activity (regular exercise or not), income 20-quantile (based on income level 4 years before baseline), residential location (metropolitan, urban, or rural), and health insurance type (employee insured, self-employed insured, or Medical Aid). Model 4: adjusted for model 3 covariates, plus body mass index (continuous), presence of abdominal obesity, hypertension, chronic kidney disease, use of statin and aspirin, blood glucose concentrations, diabetes duration (newly diagnosed type 2 diabetes, <5 years, or ≥5 years), number of oral anti-diabetic medication prescriptions per year (<3, or ≥3), and history of insulin prescription.

The associations of changes in income status between the first income assessment time point (4 years pre-baseline) and the last (baseline) with CRC risk are shown in Table 4. Individuals in the first quartile who experienced increased income had a lower risk of CRC. Interestingly, CRC risk linearly declined with increasing income rise, and those who experienced a substantial increase in income up to the top quartile had the lowest CRC risk (HR 0.77; 95% CI, 0.68–0.87, *P* for trend < 0.0001).

**Table 4.** Association between income status change (four years prior vs baseline) and colorectal cancer risk

Income status^a^ four years prior to baseline (2005–2008, first time point)	Income status^a^ at baseline (2009–2012, last time point)	Number of participants	Number of events	Total no. of person-years of follow-up	Incidence rate (per 1,000 person-years)	Risk difference (95% CI)	Adjusted HR (95% CI)
Quartile 1	Quartile 1	162,608	2,601	1,202,602	2.16	1 (Ref.)	1 (Ref.)
	Quartile 2	93,881	1,212	696,296	1.74	0.08 (0.01–0.14)	0.93 (0.87–0.99)
	Quartile 3	53,621	680	396,601	1.71	0.12 (0.04–0.19)	0.88 (0.81–0.95)
	Quartile 4	22,902	293	168,138	1.74	0.21 (0.11–0.30)	0.77 (0.68–0.87)

Quartile 2	Quartile 1	69,532	1,014	505,847	2.00	−0.06 (−0.13 to 0.00)	1.08 (1.00–1.17)
	Quartile 2	137,561	1,775	1,010,846	1.76	1 (Ref.)	1 (Ref.)
	Quartile 3	118,188	1,419	884,689	1.60	0.03 (−0.03 to 0.09)	0.97 (0.91–1.05)
	Quartile 4	28,648	393	209,853	1.87	0.04 (−0.05 to 0.13)	0.96 (0.86–1.07)

Quartile 3	Quartile 1	61,778	860	454,949	1.89	−0.07 (−0.14 to 0.00)	1.08 (1.00–1.16)
	Quartile 2	74,127	1,029	544,385	1.89	−0.07 (−0.13 to −0.01)	1.08 (1.01–1.16)
	Quartile 3	214,064	2,681	1,604,370	1.67	1 (Ref.)	1 (Ref.)
	Quartile 4	120,905	1,496	910,519	1.64	−0.01 (−0.07 to 0.04)	1.03 (0.96–1.09)

Quartile 4	Quartile 1	45,135	613	332,744	1.84	−0.01 (−0.10 to 0.06)	1.03 (0.94–1.12)
	Quartile 2	41,701	574	306,873	1.87	−0.03 (−0.11 to 0.04)	1.05 (0.96–1.15)
	Quartile 3	66,712	970	490,861	1.98	−0.03 (−0.09 to 0.03)	1.05 (0.98–1.13)
	Quartile 4	341,249	4,368	2,546,578	1.72	1 (Ref.)	1 (Ref.)

CI, confidence interval; HR, hazard ratio.

^a^Quartile 1 represents the lowest income category, while Quartile 4 represents the highest income category.

Adjusted for age, sex, pack-years of smoking (never, <10, 10 to 19, or ≥20 years), alcohol consumption (never, mild to moderate, or heavy), physical activity (regular exercise or not), income 20-quantile (based on income level 4 years before baseline), residential location (metropolitan, urban, or rural), health insurance type (employee insured, self-employed insured, or Medical Aid), body mass index (continuous), presence of abdominal obesity, hypertension, chronic kidney disease, use of statin and aspirin, blood glucose concentrations, diabetes duration (newly diagnosed type 2 diabetes, <5 years, or ≥5 years), number of oral anti-diabetic medication prescriptions per year (<3, or ≥3), and history of insulin prescription.

Stratified analyses by potential modifiers of the association between income dynamics indicators and CRC risk are shown in eTable 3. The associations of the cumulative number of years in each low- and high-income status with CRC risk were stronger among men, ever-drinkers, and ever-smokers (all *P* for interaction < 0.05). There were no other meaningful effect modifications by selected factors. The associations tended to be slightly strengthened when our analysis was limited to event-free individuals until 5 years of follow-up (eTable 4).

## DISCUSSION

In this nationwide population-based cohort study of over 1.6 million adults with T2DM, the risk of CRC was increased by 11% for those with a persistent low-income status and 10% for those with more than one income decline over 5 years, whereas living in a persistent high-income state during this period was associated with a 19% decreased risk of CRC, after accounting for multiple sociodemographic and CRC risk factors, and T2DM duration and treatment. The inverse association between persistent high-income state and CRC risk varied by anatomic site of CRC, with distal colon and rectal cancers having stronger associations than proximal colon cancer. Among individuals with the low-income status in the 4 years before baseline, CRC risk decreased linearly with increasing income levels.

Most of the prior evidence on the association between SES and CRC risk was based on one-time measurement of SES, and the findings are inconclusive.^8^^,^^9^ Differences in findings could be due to health system variability, CRC screening participation levels, and variability in other health behaviors.^8^^,^^9^ Some recent studies have investigated the association between group- or area-level income status over time and CRC risk,^15^^,^^17^^–^^21^ observing that higher or lower group-level household income was associated with decreased or increased risk of CRC, respectively, in specific time periods^17^^,^^18^ and age groups.^20^ However, the use of group- or area-level income data may not represent individual income and be subject to the ecological fallacy, making the comparison to our findings difficult.^40^ This work adds to a growing body of literature by using individual-level income data across 5 years and incorporating a rich set of covariates, including CRC risk factors, behavioral and lifestyle characteristics, and diabetic outcomes and treatment utilization.

The association between income disparities and CRC risk may be explained by multiple factors, including 1) an unhealthy lifestyle, such as physical inactivity, smoking, high alcohol consumption, and an unhealthy diet; or 2) health inequities, such as unequal access to CRC screening or prevention services.^5^^,^^6^^,^^10^ Persistent high- or low-income status in our study could better reflect such lifestyle and access factors than income measured at a single point in time. However, higher SES is not necessarily related to a healthy lifestyle, as our findings indicate that those with higher incomes smoked and drank more. This relationship also varies across cultures and the country’s development level.^41^ The association may differ between study populations and time periods, even within the same country. For instance, two South Korean studies comparing NHIS participant data from 2001 and 2009 showed the opposite associations between income exposure assessed at one point in time and colon cancer in women.^12^^,^^13^ A potential reason for this discrepancy could be the introduction of nationwide CRC screening in 2004, with no charges for low-income individuals. This policy change may have narrowed inequalities in CRC screening participation between high- and low-income groups.^42^^,^^43^

In our study, the inverse association between cumulative years of high-income status and CRC risk was stronger for distal colon and rectal cancers. This finding is consistent with previous studies, indicating a differential association between SES and CRC risk by anatomic sites.^37^^,^^38^ It has been suggested that people with higher SES have higher rates of CRC screening^44^^,^^45^ and that CRC screening is more effective in reducing the risk of left-sided CRC.^46^ Despite the nationwide expansion of public cancer screening in South Korea, inequalities in CRC screening persist, since payment and utilization for cancer screening services, particularly for opportunistic screening with colonoscopy, depend on individual income.^43^

Our findings have public health implications. T2DM is an established risk factor for CRC,^1^^,^^2^^,^^47^ and individuals with T2DM typically develop CRC almost 5 years earlier than the general population.^48^ Those with T2DM are encouraged to undertake cancer screening^4^; however, CRC screening rates in the T2DM population are often comparable to the general population or even suboptimal. Individuals with T2DM may have deficient preventive health care due to competing priorities in disease management, where income status plays a substantial role.^23^^,^^49^ Thus, understanding the impact of income dynamics and the associated factors on CRC risk is crucial for guiding efforts to reduce CRC risk among those with T2DM.

To the best of our knowledge, this is the first study to investigate the relationship between multi-year measures of income exposure and CRC risk in individuals with T2DM. In addition, we considered important confounders and effect modifiers using data from a large sample size of over 1.6 million individuals with T2DM. However, several limitations should be noted. Some potential confounders, such as a family history of CRC, dietary intake, and history of CRC screening, were unavailable. Additionally, health insurance premiums may not fully capture accurate income levels, especially regarding factors such as property holdings or unreported income for self-employed people. Furthermore, our data did not account for family dependents or household size. Moreover, using 20 quantiles of health insurance premiums as a direct ratio measure of income may not fully capture the magnitude of income changes due to their ordinal nature. Lastly, the results may not be generalizable to other populations with different socioeconomic and cultural backgrounds.

In conclusion, persistent low-income status and income declines over a 5-year window were associated with an increased risk of CRC, whereas persistent high-income status was associated with decreased risk of CRC in a sample of over 1.6 million South Korean adults with T2DM. Our data provide a better understanding of how multi-year income exposures could affect CRC risk in individuals with T2DM. Our findings underscore the need for increased public awareness about CRC risk in adults with T2DM who live in persistent low-income households or experience income declines. These insights may be important for implementing healthcare strategies and programmatic outreach, as well as for determining which populations these efforts should target.

## SUPPLEMENTARY MATERIAL

eTable 1. Distribution of baseline characteristics by income status in the baseline year^a^

eTable 2. Association of cumulative number of low- and high-income status with colorectal cancer risk in adults with type 2 diabetes

eTable 3. Association between income dynamics indicators and colorectal cancer risk in adults with type 2 diabetes stratified by selected factors

eTable 4. Association between income dynamics indicators and colorectal cancer risk in adults with type 2 diabetes: A 5-year landmark analysis
