# Supplementary material for: Income Dynamics and Risk of Colorectal Cancer in Individuals With Type 2 Diabetes: A Nationwide Population-based Cohort Study
Source: J Epidemiol. 2025 Jan 5;35(1):30–8. doi: 10.2188/jea.JE20230310 (PMC11637811; doi:10.2188/jea.JE20230310)
Supplement: Supplementary file 1 [file je-35-030-s001.pdf]

eTable 1. Distribution of baseline characteristics by income status in the baseline year[a]

	Baseline income status			
	Quartile 1	Quartile 2	Quartile 3	Quartile 4
N	339,053	347,270	452,585	513,704
**Percent (%)**				
Sex				
Male	58.6	64.8	67.3	69.9
Female	41.4	35.2	32.7	30.2
Age group, years				
<45	7.3	12.0	13.3	6.2
45-<55	47.4	48.6	47.1	54.4
≥55	45.2	39.5	39.7	39.4
Health insurance type				
Self-employed insured	19.3	30.4	34.8	40.0
Employee insured	80.7	69.6	65.2	60.0
Residential place				
Metropolitan	60.1	57.8	57.8	61.7
Urban	29.1	30.2	30.3	28.6
Rural	10.9	12.0	11.9	9.7
Smoking, pack-years				
Never	53.4	48.6	47.7	48.5
<10	9.3	11.7	12.0	11.3
10-<20	11.6	14.1	15.0	14.5
≥20	25.7	25.7	25.3	25.7
Alcohol consumption				
Non	53.6	49.1	48.0	46.7
Mild to moderate	35.4	38.0	39.0	40.7
Heavy	11.1	13.0	13.0	12.6
Regular exercise, yes[b]	19.4	19.1	19.9	23.8
Body mass index, kg/m^2				
<18.5	1.5	1.4	1.1	0.8
18.5-<23	24.9	24.7	23.0	21.3
23-<25	24.2	24.0	24.4	25.6
25-<30	41.0	41.1	42.8	44.6
≥30	8.4	8.8	8.7	7.6

Abdominal obesity, yes	36.0	35.0	36.3	36.3
Hypertension, yes	50.7	48.9	48.1	48.2
Hypercholesterolemia, yes	39.0	37.6	38.3	40.9
Chronic kidney disease, yes	6.9	6.2	6.7	7.8
Statin use, yes	26.2	25.0	25.6	28.8
Aspirin use, yes	23.0	21.3	21.6	23.5
Oral anti-diabetic medications prescribed per year (at least 3)	12.8	12.4	12.3	12.5
Insulin treatment, yes	6.4	6.4	6.2	6.2
Type 2 diabetes duration				
Newly diagnosed	49.3	51.1	51.1	47.4
<5 years	25.7	25.4	25.0	26.4
≥5 years	25.1	23.6	23.9	26.2
**Mean (standard deviation)**				
Age, years	52.6 (8.2)	51.1 (8.9)	50.9 (9.1)	52.0 (7.9)
Body mass index, kg/m^2	25.1 (3.5)	25.2 (3.5)	25.3 (3.4)	25.3 (3.2)
Waist circumference, cm	84.9 (8.8)	85.0 (8.8)	85.5 (8.6)	85.7 (8.3)
Fasting glucose, mg/dL	142.1 (52.6)	142.3 (51.8)	140.3 (49.7)	139.2 (45.7)
Systolic blood pressure, mm Hg	128.4 (16.1)	128.4 (15.9)	128.2 (15.5)	127.3 (15.0)
Diastolic blood pressure, mm Hg	79.6 (10.5)	79.8 (10.5)	79.7 (10.3)	79.3 (10.1)
Total cholesterol, mg/dL	200.0 (42.7)	199.8 (42.3)	200.0 (42.0)	198.4 (41.5)

Data are presented as percentages for categorical variables and means (standard deviation) for continuous variables.

[a] The baseline year was determined by the year in which the study subject underwent their initial health screening examination, which ranged from 2009 to 2012.

[b] Regular exercise was defined as at least 30 minutes of moderate-intensity physical activity for 5 or more days per week, or at least 20 minutes of strenuous physical activity for 3 or more days per week.

eTable 2. Association of cumulative number of low- and high-income status with colorectal cancer risk in adults with type 2 diabetes

	Number of participants	Number of events	Total number of person-years of follow-up	Incidence rate (per 1,000 person-years)	Risk difference (95% CI)	Model 1 HR (95% CI)	Model 2 HR (95% CI)	Model 3 HR (95% CI)	Model 4 HR (95% CI)
Cumulative numbers of being in low-income group									
0	1,025,648	13,153	7,646,477	1.72	1 (Ref.)	1 (Ref.)	1 (Ref.)	1 (Ref.)	1 (Ref.)
1	203,346	2,671	1,492,867	1.79	0.02 (-0.02 to 0.06)	1.04 (1.00–1.09)	1.05 (1.00–1.09)	1.03 (0.98–1.07)	1.02 (0.98–1.07)
2	133,237	1,793	982,153	1.83	0.03 (-0.01 to 0.08)	1.06 (1.01–1.12)	1.07 (1.01–1.12)	1.04 (0.99–1.10)	1.04 (0.98–1.09)
3	98,794	1,390	727,478	1.91	0.05 (-0.01 to 0.10)	1.11 (1.05–1.18)	1.09 (1.03–1.15)	1.06 (1.00–1.12)	1.06 (1.00–1.12)
4	79,304	1,119	584,514	1.91	0.01 (-0.04 to 0.07)	1.11 (1.05–1.18)	1.06 (0.99–1.12)	1.02 (0.96–1.09)	1.02 (0.95–1.09)
5	112,283	1,852	832,663	2.22	0.09 (0.03–0.07)	1.29 (1.23–1.36)	1.17 (1.11–1.23)	1.11 (1.05–1.18)	1.11 (1.04–1.18)
P for trend						<0.0001	<0.0001	0.03	0.03
Cumulative numbers of being in high-income group									
0	1,481,509	19,793	11,003,701	1.80	1 (Ref.)	1 (Ref.)	1 (Ref.)	1 (Ref.)	1 (Ref.)
1	54,726	772	403,607	1.91	0.00 (-0.06 to 0.06)	1.06 (0.99–1.14)	0.97 (0.90–1.04)	1.00 (0.93–1.07)	1.00 (0.93–1.08)
2	31,993	389	236,490	1.64	-0.14 (-0.21 to -0.06)	0.92 (0.83–1.01)	0.81 (0.73–0.90)	0.84 (0.76–0.93)	0.84 (0.76–0.93)
3	24,681	310	182,767	1.70	-0.12 (-0.21 to -0.04)	0.94 (0.84–1.06)	0.82 (0.74–0.92)	0.85 (0.76–0.96)	0.86 (0.77–0.96)
4	20,969	249	154,285	1.61	-0.16 (-0.25 to -0.07)	0.90 (0.79–1.02)	0.78 (0.69–0.89)	0.82 (0.72–0.93)	0.82 (0.72–0.93)
5	38,734	465	285,302	1.63	-0.18 (-0.24 to -0.10)	0.91 (0.83–0.99)	0.76 (0.70–0.84)	0.80 (0.73–0.88)	0.80 (0.73–0.88)
P for trend						0.01	<0.0001	<0.0001	<0.0001

CI, confidence interval; HR, hazard ratio.
Model 1: unadjusted. Model 2: adjusted for age and sex. Model 3: adjusted for model 2 covariates, plus pack-years of smoking (never, <10, 10 to 19, or ≥20 years), alcohol consumption (never, mild to moderate, or heavy), physical activity (regular exercise or not), income 20-quantile (based on income level 4 years before baseline), residential location (metropolitan, urban, or rural), and health insurance type (employee insured, self-employed insured, or Medical Aid). Model 4: adjusted for model 3 covariates, plus body mass index (continuous), presence of abdominal obesity, hypertension, chronic kidney disease, use of statin and aspirin, blood glucose concentrations, diabetes duration (newly diagnosed type 2 diabetes, <5 years, or ≥5 years), number of oral anti-diabetic medication prescriptions per year (<3, or ≥3), and history of insulin prescription.

eTable 3. Association between income dynamics indicators and colorectal cancer risk in adults with type 2 diabetes stratified by selected factors

	Association of consecutive low-income status<br><br>Adjusted HR (95% CI)	*P* for interaction	Association of consecutive high-income status<br><br>Adjusted HR (95% CI)	*P* for interaction	Association of number of income declines (≥2)<br><br>Adjusted HR (95% CI)	*P* for interaction	Association of high income (quartile 4) at baseline<br><br>Adjusted HR (95% CI)	*P* for interaction
**Age groups, years**		0.72		**0.19**		0.37		0.14
<45	1.17 (0.72–1.92)		2.07 (0.52–8.31)		1.37 (0.97–1.95)		0.91 (0.66–1.24)	
45-54	1.16 (1.05–1.27)		0.74 (0.63–0.87)		1.11 (1.02–1.21)		0.87 (0.81–0.92)	
≥55	1.07 (1.00–1.15)		0.84 (0.75–0.94)		1.09 (1.02–1.16)		0.92 (0.87–0.97)	
**Sex**		**0.005**		**0.0002**		**0.02**		**0.001**
Male	1.14 (1.07–1.22)		0.76 (0.68–0.85)		1.10 (1.03–1.16)		0.86 (0.82–0.90)	
Female	1.04 (0.94–1.14)		0.98 (0.81–1.19)		1.11 (1.01–1.21)		1.00 (0.93–1.08)	
**Smoking**		0.06		**0.01**		0.69		0.27
Never	1.06 (0.97–1.15)		0.92 (0.81–1.05)		1.09 (1.01–1.18)		0.93 (0.88–0.99)	
Ever	1.15 (1.07–1.24)		0.72 (0.63–0.82)		1.11 (1.03–1.18)		0.87 (0.83–0.92)	
**Alcohol consumption**		**<0.0001**		**<0.0001**		**0.0006**		**<0.0001**
Never	1.07 (0.99–1.16)		0.97 (0.85–1.11)		1.03 (0.96–1.11)		0.98 (0.93–1.04)	
Ever	1.14 (1.06–1.24)		0.69 (0.61–0.79)		1.16 (1.08–1.24)		0.83 (0.79–0.88)	
**Regular exercise**		0.28		0.54		0.14		0.27
No	1.13 (1.06–1.20)		0.79 (0.70–0.88)		1.13 (1.07–1.19)		0.88 (0.84–0.93)	
Yes	1.04 (0.93–1.17)		0.85 (0.72–1.01)		0.99 (0.89–1.11)		0.95 (0.88–1.03)	
**Residential location**		0.16		0.57		0.56		0.43
Metropolitan	1.16 (1.08–1.25)		0.82 (0.73–0.91)		1.09 (1.02–1.16)		0.89 (0.84–0.94)	
Urban	1.02 (0.93–1.13)		0.83 (0.69–1.00)		1.09 (0.99–1.20)		0.92 (0.85–0.99)	
Rural	1.04 (0.89–1.22)		0.60 (0.39–0.91)		1.20 (1.04–1.39)		0.91 (0.81–1.02)	
**Health insurance type**		**0.02**		0.49		0.41		0.39
Self-employed insured	1.03 (0.93–1.14)		0.79 (0.70–0.91)		1.03 (0.92–1.16)		0.92 (0.85–0.99)	
Employee insured	1.14 (1.06–1.22)		0.82 (0.72–0.93)		1.12 (1.06–1.18)		0.90 (0.86–0.95)	
**Body mass index, kg/m^2**		**0.06**		0.58		0.61		0.21
<25	1.15 (1.06–1.24)		0.84 (0.73–0.95)		1.08 (1.01–1.16)		0.90 (0.85–0.96)	
≥25	1.07 (0.99–1.16)		0.78 (0.68–0.89)		1.12 (1.04–1.20)		0.90 (0.85–0.95)	
**Type 2 diabetes duration**		0.43		0.88		0.51		0.18
New onset	1.14 (1.06–1.24)		0.80 (0.69–0.93)		1.15 (1.07–1.24)		0.88 (0.83–0.93)	
<5 years	1.09 (0.99–1.21)		0.77 (0.65–0.93)		1.08 (0.98–1.18)		0.88 (0.82–0.95)	
≥5 years	1.06 (0.96–1.18)		0.83 (0.71–0.98)		1.04 (0.95–1.15)		0.95 (0.89–1.02)	

CI, confidence interval; HR, hazard ratio.
Adjusted for age, sex, pack-years of smoking (never, <10, 10 to 19, or ≥20 years), alcohol consumption (never, mild to moderate, or heavy), physical activity (regular exercise or not), income 20-quantile (based on income level 4 years before baseline), residential location (metropolitan, urban, or rural), health insurance type (employee insured, self-employed insured, or Medical Aid), body mass index (continuous), presence of abdominal obesity, hypertension, chronic kidney disease, use of statin and aspirin, blood glucose concentrations, diabetes duration (newly diagnosed type 2 diabetes, <5 years, or ≥5 years), number of oral anti-diabetic medication prescriptions per year (<3, or ≥3), and history of insulin prescription, except for the stratifying variable.

eTable 4. Association between income dynamics indicators and colorectal cancer risk in adults with type 2 diabetes: A 5-year landmark analysis

	Number of participants	Number of events	Total number of person-years of follow-up	Incidence rate (per 1,000 person-years)	Risk difference (95% CI)	Model 1	Model 2	Model 3	Model 4
						HR (95% CI)	HR (95% CI)	HR (95% CI)	HR (95% CI)
Cumulative numbers of being in low-income group									
0	997,089	4,748	2,583,044	1.84	1 (Ref.)	1 (Ref.)	1 (Ref.)	1 (Ref.)	1 (Ref.)
1–4	496,391	2,594	1,255,434	2.07	0.04 (0.01–0.09)	1.12 (1.07–1.18)	1.11 (1.06–1.17)	1.07 (1.01–1.13)	1.07 (1.01–1.13)
5	107,324	714	282,464	2.53	0.11 (0.03–0.20)	1.38 (1.27–1.49)	1.24 (1.15–1.34)	1.15 (1.04–1.27)	1.15 (1.04–1.27)
P for trend						<0.0001	<0.0001	0.011	0.01
Number of income declines									
0	1,014,149	4,942	2,645,897	1.87	1 (Ref.)	1 (Ref.)	1 (Ref.)	1 (Ref.)	1 (Ref.)
1	468,907	2,446	1,180,751	2.07	0.04 (0.00–0.08)	1.11 (1.06–1.16)	1.07 (1.02–1.12)	1.05 (1.00–1.11)	1.05 (1.00–1.10)
≥2	117,748	668	294,294	2.27	0.12 (0.05–0.19)	1.22 (1.12–1.32)	1.17 (1.08–1.27)	1.17 (1.08–1.27)	1.16 (1.07–1.26)
P for trend						<0.0001	0.0002	0.0004	0.0008
Cumulative numbers of being in high-income group									
0	1,433,636	7,348	3,704,976	1.98	1 (Ref.)	1 (Ref.)	1 (Ref.)	1 (Ref.)	1 (Ref.)
1–4	129,246	561	322,422	1.74	-0.14 (-0.02 to -0.70)	0.88 (0.81–0.96)	0.78 (0.72–0.85)	0.82 (0.75–0.90)	0.83 (0.76–0.91)
5	37,922	147	93,544	1.57	-0.22 (-0.32 to -0.12)	0.79 (0.67–0.93)	0.67 (0.57–0.79)	0.72 (0.61–0.85)	0.72 (0.61–0.86)
P for trend						0.0003	<0.0001	<0.0001	<0.0001
Baseline income									
Quartile 1	325,941	1,936	830,757	2.33	1 (Ref.)	1 (Ref.)	1 (Ref.)	1 (Ref.)	1 (Ref.)
Quartile 2	334,948	1,721	850,051	2.02	-0.05 (0.11–0.00)	0.87 (0.81–0.93)	0.94 (0.88–1.01)	0.94 (0.88–1.00)	0.94 (0.88–1.00)
Quartile 3	438,861	2,101	1,144,752	1.84	-0.11 (-0.16 to -0.06)	0.79 (0.74–0.84)	0.86 (0.81–0.92)	0.87 (0.82–0.93)	0.87 (0.82–0.93)
Quartile 4	501,054	2,298	1,295,383	1.77	-0.15 (-0.21 to -0.09)	0.76 (0.72–0.81)	0.80 (0.75–0.85)	0.82 (0.76–0.88)	0.83 (0.77–0.89)
P for trend						<0.0001	<0.0001	<0.0001	<0.0001

CI, confidence interval; HR, hazard ratio.
Model 1: unadjusted. Model 2: adjusted for age and sex. Model 3: adjusted for model 2 covariates, plus pack-years of smoking (never, <10, 10 to 19, or ≥20 years), alcohol consumption (never, mild to moderate, or heavy), physical activity (regular exercise or not), income 20-quantile (based on income level 4 years before baseline), residential location (metropolitan, urban, or rural), and health insurance type (employee insured, self-employed insured, or Medical Aid). Model 4: adjusted for model 3 covariates, plus body mass index (continuous), presence of abdominal obesity, hypertension, chronic kidney disease, use of statin and aspirin, blood glucose concentrations, diabetes duration (newly diagnosed type 2 diabetes, <5 years, or ≥5 years), number of oral anti-diabetic medication prescriptions per year (<3, or ≥3), and history of insulin prescription.
